# Pre-immune state induced by chicken interferon gamma inhibits the replication of H1N1 human and H9N2 avian influenza viruses in chicken embryo fibroblasts

**DOI:** 10.1186/s12985-016-0527-1

**Published:** 2016-04-27

**Authors:** Seong-Su Yuk, Dong-Hun Lee, Jae-Keun Park, Erdene-Ochir Tseren-Ochir, Jung-Hoon Kwon, Jin-Yong Noh, Joong-Bok Lee, Seung-Yong Park, In-Soo Choi, Chang-Seon Song

**Affiliations:** Department of Avian Diseases Laboratory, College of Veterinary Medicine, Konkuk University, Seoul, Korea

**Keywords:** Chicken interferon gamma, Chicken embryo fibroblast, Influenza virus, Interferon stimulated genes, Pre-immune state

## Abstract

**Background:**

Interferon gamma (IFN-γ), an immunoregulatory cytokine, is known to control many microbial infections. In a previous study, chicken interferon gamma (chIFN-γ) was found to be up-regulated following avian influenza virus (AIV) infection in specific pathogen-free chickens. We aimed to investigate whether the pre-immune state induced by chIFN-γ could generate an antiviral response against influenza virus.

**Methods:**

We generated a chIFN-γ-expressing plasmid and transfected it into chicken embryo fibroblasts (CEFs) and then infected the cells with human origin H1N1 or avian origin H9N2 influenza viruses. Viral titers of culture medium were evaluated in MDCK cell and the viral RNA and IFN-stimulated genes (ISGs) were then quantified by real-time reverse transcriptase polymerase. To further evaluate the role of the antiviral effect of chIFN-γ by using a backward approach, synthetic small interfering RNAs (siRNA) targeting chIFN-γ were used to suppress chIFN-γ.

**Results:**

The chIFN-γ-stimulated CEFs inhibited the replication of viral RNA (vRNA) and showed a mild decrease in the infectious virus load released in the culture medium. Compared to the mock-transfected control, the messenger RNA (mRNA) levels of type I IFNs and IFN-stimulated genes were up-regulated in the cells expressing chIFN-γ. After treatment with the siRNA, we detected a higher expression of viral genes than that observed in the mock-transfected control.

**Conclusions:**

Our results suggest that apart from the important role played by chIFN-γ in the antiviral state generated against influenza virus infection, the pre-immune state induced by chIFN-γ can be helpful in mitigating the propagation of influenza virus.

**Electronic supplementary material:**

The online version of this article (doi:10.1186/s12985-016-0527-1) contains supplementary material, which is available to authorized users.

## Background

Influenza A virus is an enveloped virus from the *Orthomyxoviridae* family, with an eight-segmented, single-stranded, negative-sense RNA genome. It is classified into subtypes based on the combination of 16 haemagglutinin (HA) and 9 neuraminidase proteins [1]. Because of its nature as an RNA virus, influenza virus is able to modify its genome by any of the several types of mutations, including antigenic variation, natural selection, and reassortment [2]. This has resulted in continuing spread of avian influenza virus (AIV), and has caused great economic loss in the poultry industry, despite regular vaccination programs [3, 4]. The economic losses inflicted by H9N2 low-pathogenic avian influenza is, especially, an ongoing problem in domestic poultry farms in South Korea since 1996 [5].

The members of the type I interferon (IFN) family induce antiviral states by binding to a common receptor, consisting of two interferon-α/β receptor subunits (IFNAR1 and IFNAR2), on the cell membrane of most cells. JAK/STAT signaling via IFNAR1 and IFNAR2 enhances the expression of many antiviral proteins, including myxovirus-resistance protein (Mx) GTPase, RNA-dependent protein kinase (PKR), latent ribonuclease (RNaseL), and oligo-adenylate synthetase (OAS). These IFN-induced antiviral proteins play a critical role in innate, as well as adaptive, immune responses against viral infections [6]. Because of their non-specific and pattern-recognition properties, these IFN-induced antiviral proteins have an effect on various strains of the influenza virus [7, 8]. IFN-γ, which is the only type II IFN, binds to a distinct receptor comprising two IFN-γ receptor subunits (IFNGR1 and IFNGR2). The binding of IFN-γ to IFNGR induces the production of IFN regulatory factor (IRF)-9 (also known as ISGF3), via the JAK/STAT signaling pathway. The involvement of common signaling pathways means that IFN-γ can activate type I IFNs, and may explain many overlapping effects of type I and II IFNs [9].

Chicken IFN-γ (chIFN-γ), first cloned by Digby and Lowenthal, is known as a regulator of immune responses, including antiviral defenses [10]. Several evidence show that the chicken immune system following infection with AIV begin to express proinflammatory cytokines, which results in a general antiviral response through the activation of a broad range of effector molecules, including Mx, PKR, and OAS [11, 12]. We also showed that immunocompromised chickens showed elevated level of vRNA with relative down-regulation of chIFN-γ after AIV infection [13]. This finding is evidence to the fact that the regulation of proinflammatory cytokines correlates with the antiviral status. In addition, it has been shown to enhance the immune response of vaccine against Marek's disease virus and Newcastle disease virus [14, 15]. The immunity-enhancing effects of IFN-γ have also been reported in other avian species. For example, administration of recombinant IFN-γ inhibited the replication of duck hepatitis B hepatocytes [16]. Although it’s not for viral infection, administration of chIFN-γ to chicken enhanced weight gain in the face of coccidial infection, which showed a possibility for practical use of chIFN-γ [17].

On the basis of previous studies, we aimed to determine the antiviral effect of the pre-immune state against H1N1 human and H9N2 avian influenza infection in CEFs, which was investigated to evaluate the influence of chIFN-γ expression on innate immunity. Although several studies showed a correlation between AIV infection and type I IFNs, few studies have investigated the antiviral effects of IFN-γ against influenza infection at the cellular level [18]. In addition, we compared the kinetics of antiviral gene expression following influenza infection to evaluate the effect of modulation of chIFN-γ on type I IFN and IFN-induced antiviral genes.

## Methods

### Cells and virus

Madin-Darby canine kidney (MDCK) cells (ATCC, CRL-34) were used to measure the viral titer of the CEF medium. MDCK cells were maintained as a monolayer culture in minimum essential medium (MEM) supplemented with 10 % fetal bovine serum (FBS) and 1 % penicillin/streptomycin, at 37 °C with 5 % CO_2_. CEF cultures were prepared from 10-day-old specific pathogen-free (SPF) embryonated eggs, following established protocols [19]. CEFs were cultured in M199 (GIBCO, Grand Island, N.Y.) and Ham's F-10 (Sigma, St Louis, MO, USA) mixed medium, containing 10 % bovine serum (BS), 0.65 % sodium bicarbonate, and 1 % penicillin/streptomycin. H9N2 avian influenza virus (A/Korean native chicken/Korea/K040110/2010) and H1N1 human influenza virus (A/NWS/1933) were propagated in 10-day-old SPF embryonated eggs and kept frozen at −80 °C for further use.

### Plasmid construction

Chicken peripheral blood mononuclear cells (PBMCs) were purified using Histopaque 1077 (Sigma) gradient separation. The cells were grown overnight in 3 ml of Roswell Park Memorial Institute (RPMI) medium supplemented with 5 % FBS, 1 % penicillin/streptomycin, and 10 μl of 2.5 mg/ml concanavalin A. Total RNA was isolated from PBMCs by using an RNeasy mini kit (Qiagen, Hilden, Germany), and was used as a template for cDNA synthesis using an Omniscript RT kit (Qiagen). The chIFN-γ coding sequence was amplified using primers designed to span the predicted chIFN-γ-coding sequence, which was obtained from NCBI database (GenBank accession no. AY501004.1). The amplified PCR product was cloned into the pcDNA3.1(+) expression vector (Invitrogen, Darmstadt, Germany), amplified, and purified using a Plasmid Maxi kit (Qiagen).

### Transfection and detection of chIFN-γ

Briefly, 6 × 10^5^ cells per well were seeded in 6-well culture plate and incubated at 37 °C for 24 h. The CEFs (about 2 × 10^6^ cells) were transfected with 0.5 μg of pcDNA3.1(+) or pcDNA-chIFN-γ using Lipofectamine 2000 (Invitrogen), and the medium was harvested at 6, 12, 24, and 48 h post transfection. The medium samples were then subjected to diafiltration, using 10-kDa molecular weight cut-off Vivaspin spin columns (Sartorius Stedim Biotech, Goettingen, Germany), at 10,000 × *g* at 4 °C. The concentrated culture medium samples were resolved by sodium dodecyl sulfate-polyacrylamide gel electrophoresis, and analyzed by western blotting using an anti-chIFN-γ rabbit polyclonal antibody (Kingfisher Biotech, St Paul, MN, USA) and peroxidase-conjugated anti-rabbit goat monoclonal secondary antibody (Sigma). Immunofluorescence staining was also performed to evaluate the expression of chIFN-γ. Transfected CEFs were grown on 24-well plates for 1 day and fixed with 4 % paraformaldehyde in phosphate-buffered saline (PBS) for 10 min at room temperature (20 to 25 °C). The cells were permeabilized with 0.5 % Triton X-100 in PBS for 7 min and washed three times with a washing solution containing 10 % FBS and 0.2 % Tween-20 in PBS. Then, the cells were incubated with anti-chIFN-γ rabbit polyclonal antibody (Kingfisher Biotech) for 1 h at room temperature. After washing, the secondary antibodies conjugated with Alexa Fluor 488 goat anti-rabbit immunoglobulin G (Invitrogen) were applied. After staining with 4′,6-diamidino-2-phenylindole (Sigma), the fluorescence images were acquired using an AxioVert 200 inverted-microscope (Carl Zeiss, Germany). The percentage of fluorescent cells was calculated from following formula: % transfection efficacy = fluorescent cell green fluorescent cell number / blue fluorescent cell number × 100.

### Virus infection of transfected CEFs

CEFs transfected with pcDNA3.1(+) or pcDNA-chIFN-γ were prepared 24 h before infection. Transfected CEFs were infected with H1N1 or H9N2 virus at a multiplicity of infection (MOI) of 0.01, which was diluted in the CEF medium at 37 °C with gentle agitation every 15 min. After 1 h of incubation, the unabsorbed virus was removed, and the cells were washed with PBS. Fresh medium supplemented with 1 μg/ml tosylsulfonyl-phenylalanyl-chloromethyl ketone-treated trypsin (Sigma) was added, and the plates were incubated at 37 °C in with 5 % CO_2_. At 0, 6, 12, 24, and 48 h post infection (hpi), the supernatant and CEFs were separated, harvested, and stored at −80 °C for virus titration and vRNA quantification.

### Virus titration

MDCK cells were used to determine the viral titer of propagated influenza virus to verify the biological activity of the released infectious virion particles. The released virion particles accumulate in the culture medium and can be quantified in the medium. A confluent 96-well culture plate of MDCK cells was prepared at 24 h before undertaking the virus titration (TCID_50_) assay. The cells were washed once with PBS, and the medium was replenished with serum-free MEM medium supplemented with 100 units/ml penicillin, 100 μg/ml streptomycin, and 2 μg/ml trypsin. Serial dilutions of the supernatant, from 1 log to 8 log, were performed, and each dilution was added to eight wells of a 96-well culture plate in triplicate. The plates were then observed daily for cytopathic effects (CPE). The end-point of viral dilution leading to CPE in 50 % of the inoculated wells was estimated using the Karber method [20].

### Quantification of vRNA and cytokine mRNA

For relative quantification of vRNA and cytokine mRNA in CEFs, total RNA was extracted from virus-infected CEFs samples by using RNeasy Mini Kit (Qiagen), according to the manufacturer’s instructions. The vRNA and IFN-stimulated genes (ISGs) were then quantified by real-time reverse transcriptase polymerase chain reaction (rRT-PCR) in a thermal cycler (Smart Cycler System, Cepheid, Sunnyvale, CA, USA) using One-Step SYBR Green Master Mix II (Takara, Dalian, Jiangsu, China) according to the manufacturer’s instructions. The primer sets for hemagglutinin (HA), polymerase basic 2 (PB2) genes, chicken IFN-α, chicken IFN-β, chicken myxovirus resistant 1 (chMx), chicken 2'-5' oligoadenylate synthetase (chOAS), chicken RNase L, and chicken protein kinase R (chPKR) mRNAs are listed in Additional file 1: Table S1. The vRNA was quantified and compared to that in the mock-transfected control. The expression of cytokines and ISGs was normalized using the comparative 2^-2∆∆*Ct*^ method, which was used to determine the mean fold increase in the expression level of the respective gene from the corresponding time point in the uninfected cell [21].

### Treatment with siRNA targeting chIFN-γ

SiRNA transfection, at a final concentration of 50 nM, was carried out using Lipofectamine 2000 (Invitrogen) in CEFs. An siRNA targeting chIFN-γ [chIFN-γ-siRNA 277 bp: 5'-CCGCACAUCAAACACAUAU(dTdT)-3'] and a validated negative control siRNA [Negative control: 5'-CCUACGCCAAUUUCGU-3'] were designed and synthesized by ST Pharm Co., Ltd. Because we could not differentiate mock-transfected CEF from the chIFN-γ-siRNA transfected CEF using immunofluorescence assay, the knockdown of chIFN-γ mRNA was confirmed by the real-time RT-PCR. The primer set for chIFN- γ is listed in Additional file 1: Table S1.

The CEFs were inoculated with H1N1 and H9N2 virus at an MOI of 0.01 at 12 h after transfection. CEF culture medium was harvested 48 hpi, and RNA was extracted for rRT-PCR analysis. To compare viral gene replication within CEFs, the vRNA was quantified by rRT-PCR as described above.

### Statistical analyses

Data are expressed as the mean and standard deviation. Statistical differences for time related cytokine expression after transfection of pcDNA3.1(+) or pcDNA-chIFN-γ were analyzed with two-way ANOVA using Prism 5 (GraphPad Co., San Diego, CA, USA). To compare viral RNA difference between pcDNA3.1(+) and pcDNA-chIFN-γ, unpaired student *t*-test was used. *P* values < 0.05 were considered significant.

## Results

### Cloning and expression of chIFN-γ

**Fig. 1:**
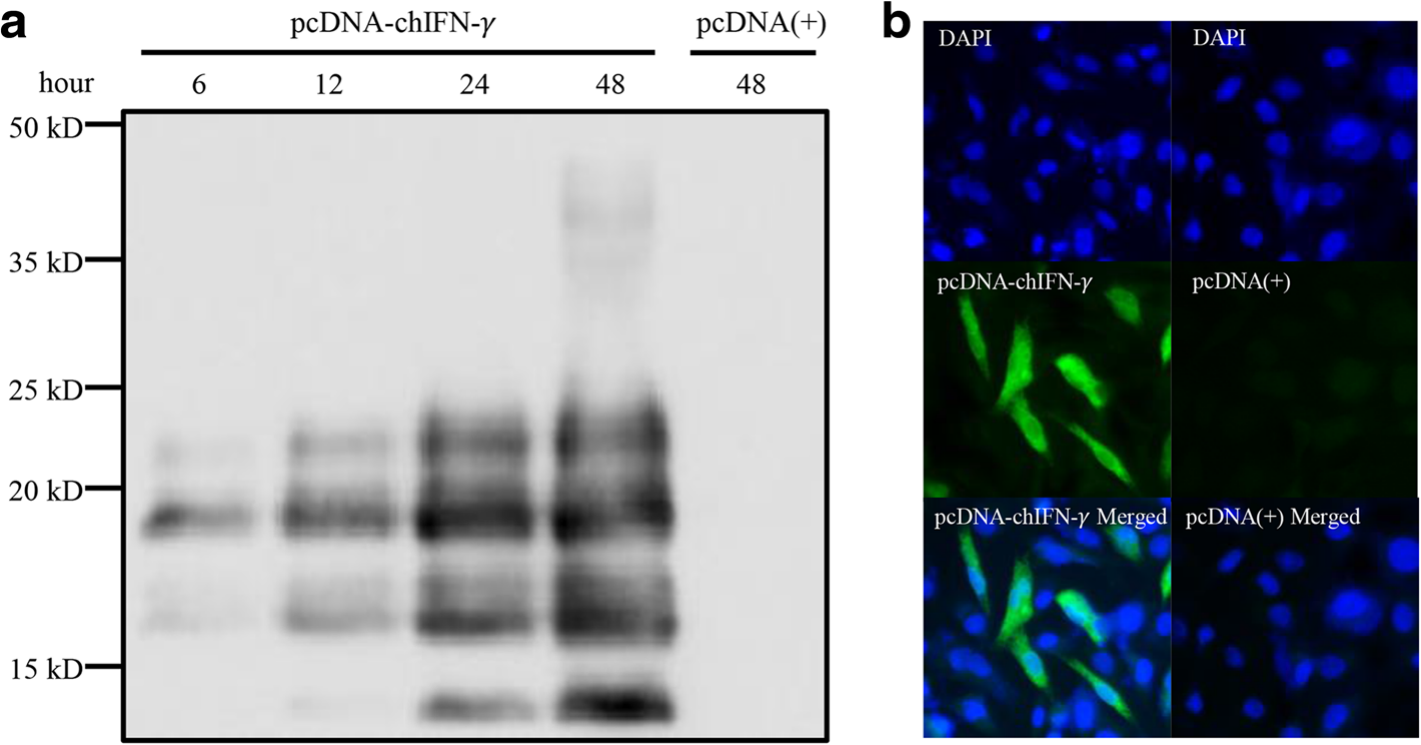
Expression of chIFN-γ in CEFs and the cell culture medium. **a** CEFs were transfected with pcDNA-IFN-γ. Then, the cell culture medium was harvested at 6, 12, 24, and 48 h post transfection. The medium was centrifuged and concentrated using spin columns. Western blotting was performed as described in Methods. **b** At 24 h post transfection, the localization of chIFN-γ in CEFs was demonstrated using anti-chIFN-γ polyclonal rabbit antibody as the primary antibody and anti-rabbit IgG Alexa Fluor 488 goat antibody as the secondary antibody (green). Cell nuclei were stained with 4,6-diamidino-2-phenylindole (blue)

Western blotting revealed the presence of 13–24-kDa bands corresponding to chIFN-γ in the culture medium of pcDNA-chIFN-γ-transfected CEFs. The bands detected were of 3–4 different sizes, rather than a single band of the calculated size (16.7 kDa), which corresponded to its theoretical molecular weight. Major bands were noted at 17 and 19 kDa, and weak bands were observed above them. Although the samples were reduced by 5 % 8-mercaptoethanol, we suppose that a dimeric form of chIFN-γ was also observed at 48 h after transfection in minor quantities which was not reduced. The intensity of the bands increased, indicating higher levels of expression, with time. No bands were detected in the medium containing the mock-transfected control cells (Fig. 1a). The expression of chIFN-γ in CEFs transfected with pcDNA-chIFN-γ was also verified by an immunofluorescence assay using an anti-chIFN-γ polyclonal antibody (Fig. 1b), whereas the mock-transfected control cells did not emit any fluorescence with the anti-chIFN-γ polyclonal antibody. The percentage of fluorescent cells was calculated from the image and transfection efficiency of pcDNA-chIFN-γ was about 25%. These results not only indicate that chIFN-γ was expressed successfully in CEFs but also that pcDNA3.1(+)-transfected CEFs do not release detectable levels of chIFN-γ in the culture medium or within the CEFs.

### Inhibition of AIV replication in the CEF culture medium

**Fig. 2:**
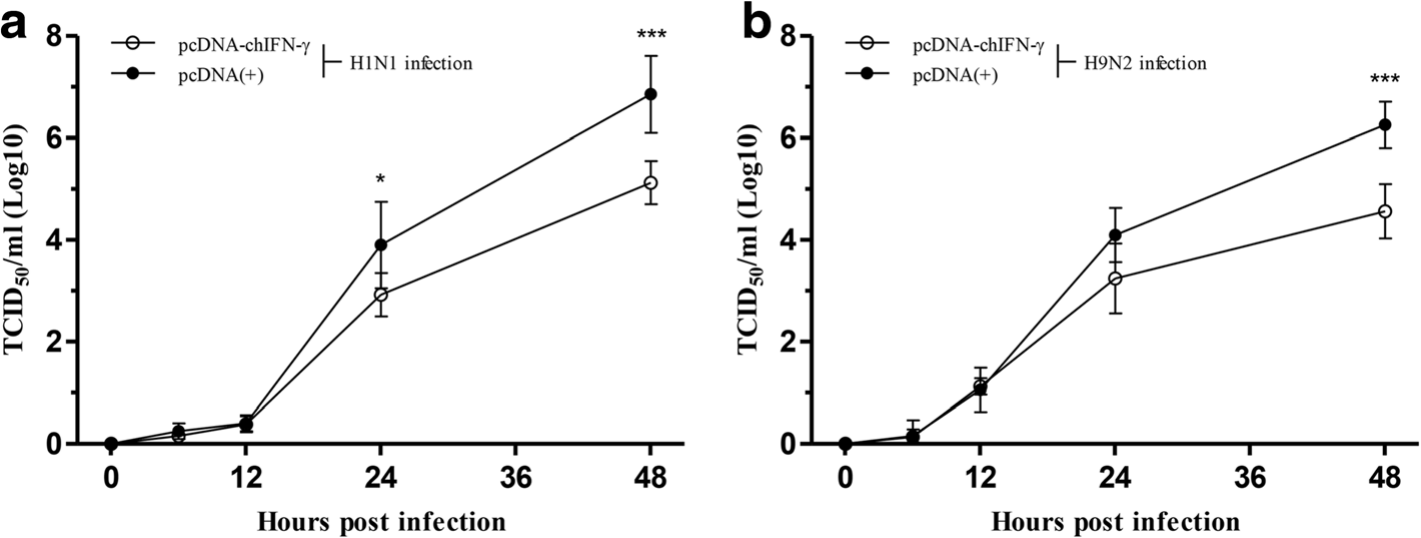
Reduced viral titer of H1N1 and H9N2 influenza viruses in CEF expressing chIFN-γ. Twenty-four hours after transfection with pcDNA-IFN-γ, CEFs were infected with H1N1 and H9N2 virus. Forty-eight hours post infection, MDCK cells were treated with **a** H1N1- and **b** H9N2-infected CEF culture supernatants. TCID_50_ titer was measured by end-point dilution assay. Statistical significance was determined by one-way ANOVA (*P < 0.05, **P < 0.01, and ***P < 0.001)

To measure the amount of infectious viral particles that were released into the culture supernatant, a TCID_50_ titration assay was performed by end-point dilution assay. The CEFs transfected with pcDNA-chIFN-γ showed lower levels of viral titer than did the cells transfected with pcDNA3.1(+) after H1N1 and H9N2 virus infection. There were no differences in the titer during the early phases of replication in both H1N1 and H9N2 virus infection. However, lower level of viral titer was observed after 24 hpi with chIFN-γ. At 24 and 48 hpi, the mean viral titer was significantly reduced by approximately 10 and 60 folds against H1N1 infection (Fig. 2a). In H9N2 infection, no statistical reduction in virus titer was observed at 6, 12, and 24 hpi. At 48 hpi, approximately 50-fold reduction in viral titer was observed in H9N2 infection (Fig. 2b). These data demonstrate that although the decrease was somewhat mild, the CEFs stimulated by chIFN-γ ahead of infection reduce virus replication in H1N1 and H9N2 infection.

### Inhibition of AIV replication in CEF

**Fig. 3:**
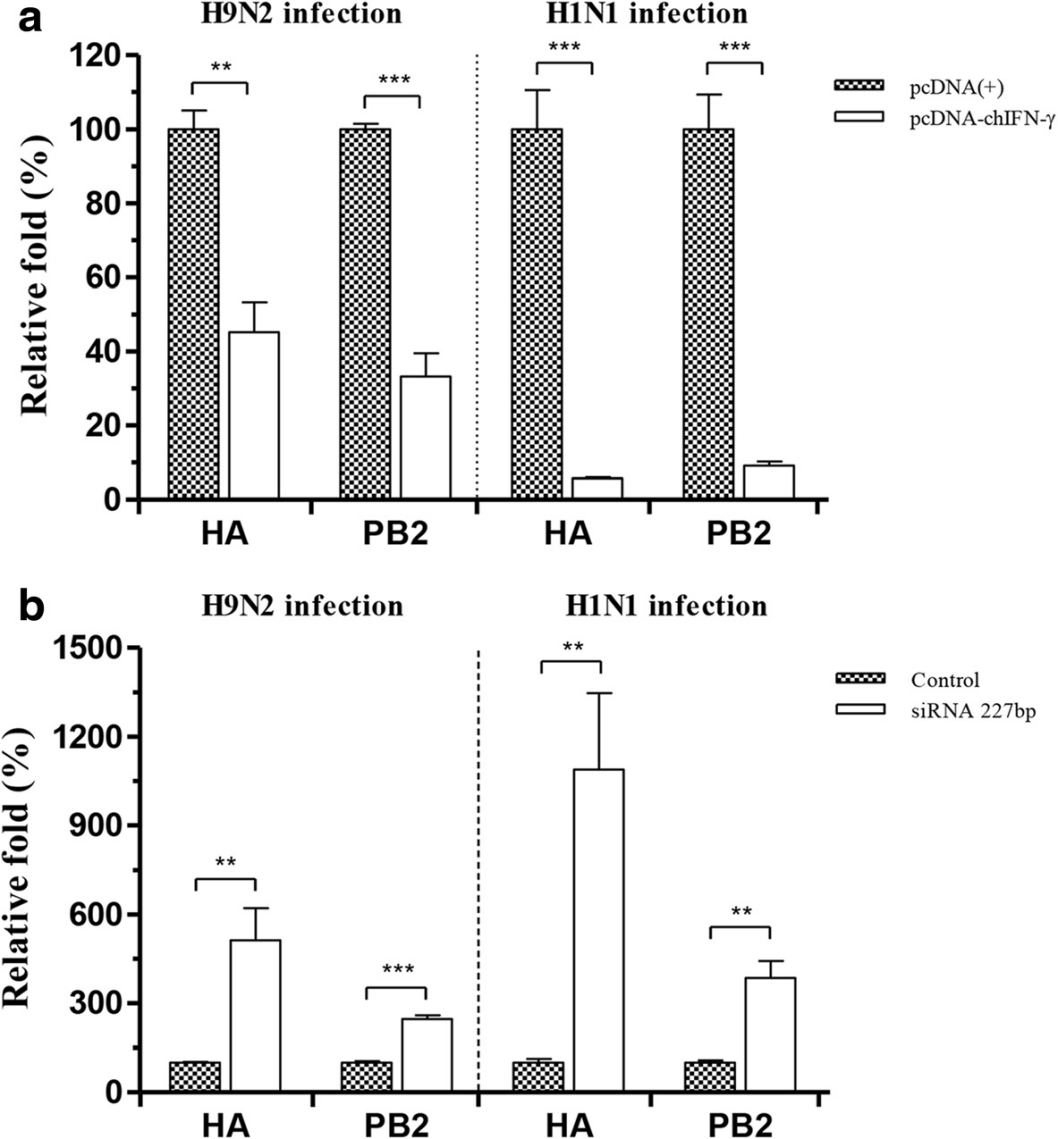
Viral gene replication in CEF expressing chIFN-γ and suppressed by siRNA targeting chIFN-γ. CEFs were harvested and analyzed by real-time RT-PCR after H1N1 and H9N2 virus infection. **a** The mRNAs of viral glycoprotein (HA) and viral polymerase (PB2) were significantly reduced in pcDNA-chIFN-γ-transfected CEFs compared to the control. **b** HA and PB2 mRNA was significantly increased in siRNA targeting chIFN-γ transfected CEFs compared to the control. Statistical significance was determined by unpaired student *t*-test (*P < 0.05, **P < 0.01, and ***P < 0.001)

Forty-eight hours after infection, the cells were harvested and analyzed by rRT-PCR. As compared to the mock-transfected control, transfection with pcDNA-chIFN-γ significantly reduced the fold change in viral RNA levels after infection, from 2.2- to 17-fold (Fig. 3a). These results suggest that both early (PB2) and late (HA) transcribed genes were down-regulated in CEFs transfected with chIFN-γ. Although the reduction of viral titer which were measured in culture medium was approximately same in both viruses at 48 hpi, reduction of viral gene within the CEFs was more potent against H1N1 than H9N2 infection.

### Effects of chIFN-γ-siRNA transfection on AIV replication in CEFs

To investigate whether the down-regulation of chIFN-γ resulted in the replication of H1N1 and H9N2 genes, the siRNAs (chIFN-γ-siRNA 277 bp) were designed to specifically target chIFN-γ mRNA. The expression levels of chIFN-γ mRNA in the CEF culture medium were successfully reduced by transfection with chIFN-γ-siRNA 277 bp [see Additional file 2]. When the cells were transfected with the siRNA, the CEFs showed high levels of HA and PB2 gene replication after infection; the levels were significantly high, compared to the control (Fig. 3b). This suggests that the suppression of chIFN-γ, which was regulated before the infection, results in increased replication of viral genes.

### Regulation of ISGs by chIFN-γ

**Fig. 4:**
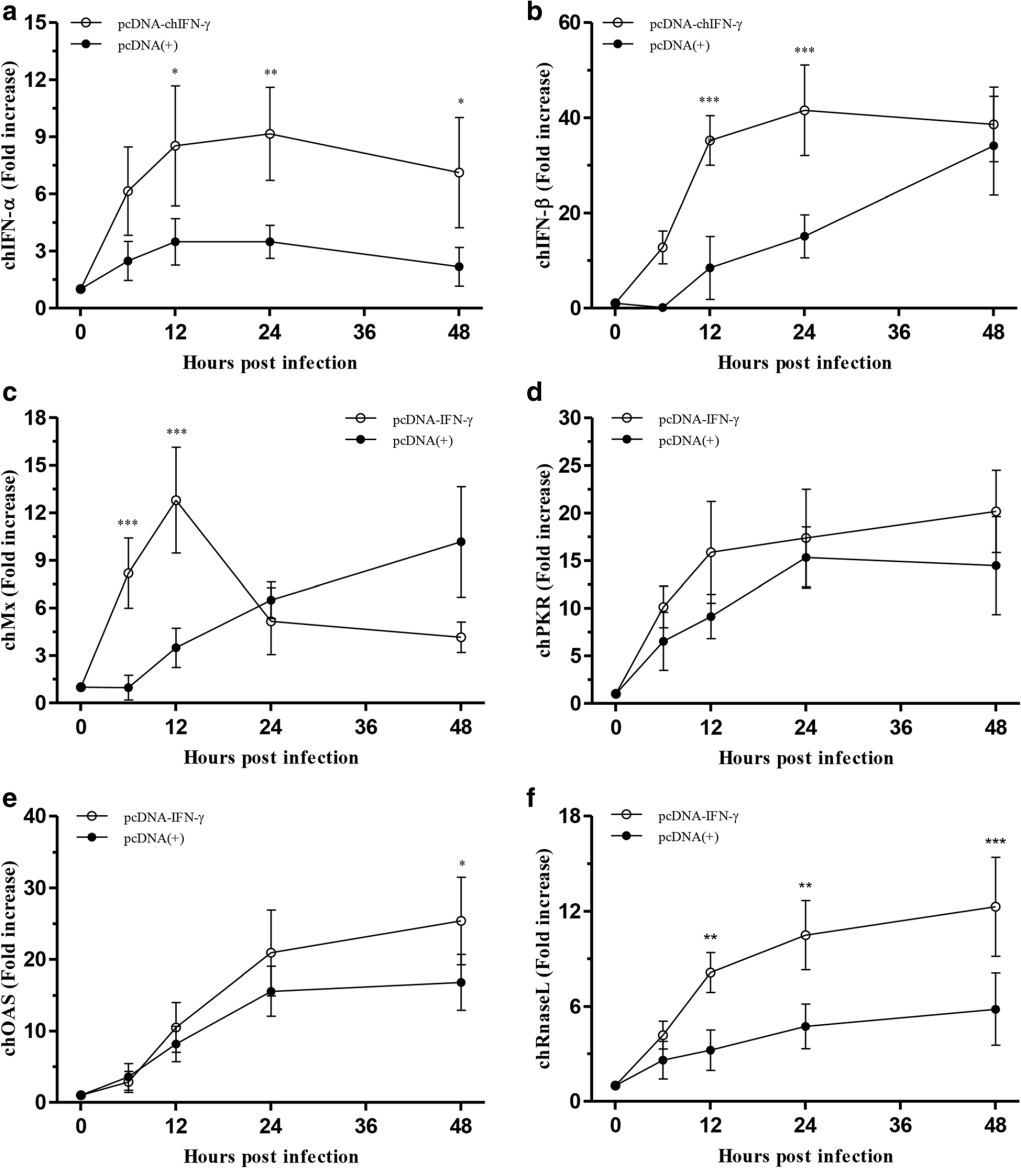
Relative expression of type I interferons and interferon-stimulated genes following H1N1 infection in CEFs. Twenty-four hours after transfection with pcDNA-IFN-γ, CEFs were infected with 0.01 MOI of H1N1 virus. At 0, 6, 12, 24, and 48 hpi, the total RNA content of CEFs was extracted and analyzed by real-time RT-PCR, as described in Methods. The expression levels of chIFN-α (**a**), chIFN-β (**b**), chMx (**c**), chPKR (**d**), and chOAS (**e**), and chRNaseL (**f**) were assessed. Statistical significance was determined by two-way ANOVA (*P < 0.05, **P < 0.01, and ***P < 0.001)

**Fig. 5:**
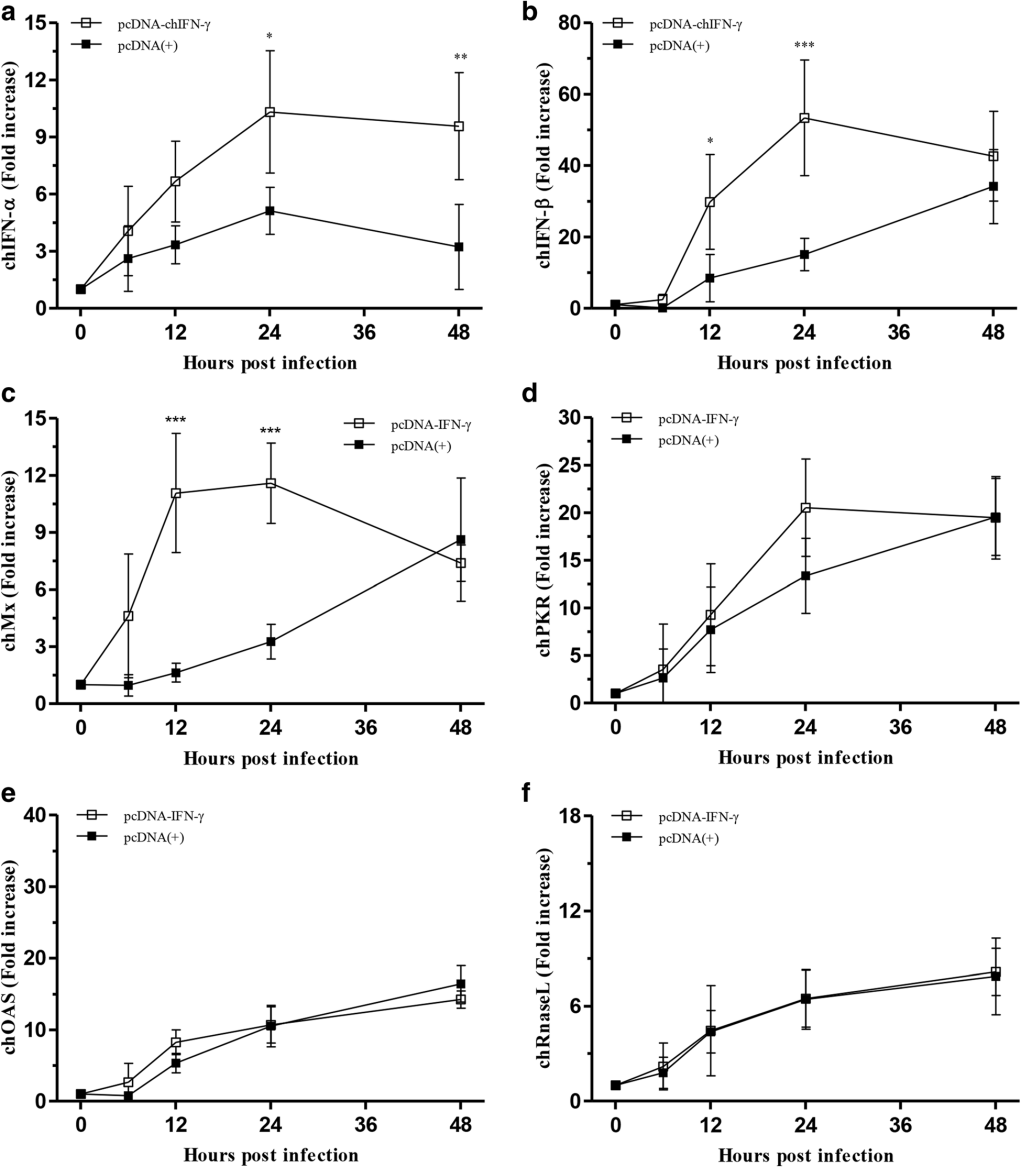
Relative expression of type I interferons and interferon-stimulated genes following H9N2 infection in CEFs. Twenty-four hours after transfection with pcDNA-IFN-γ, CEFs were infected with 0.01 MOI of H9N2 virus. At 0, 6, 12, 24, and 48 hpi, the total RNA content of CEFs was extracted and analyzed by real-time RT-PCR, as described in Methods. The expression levels of chIFN-α (**a**), chIFN-β (**b**), chMx (**c**), chPKR (**d**), and chOAS (**e**), and chRNaseL (**f**) were assessed. Statistical significance was determined by two-way ANOVA (*P < 0.05, **P < 0.01, and ***P < 0.001)

To study the regulation of ISGs after H1N1 and H9N2 virus infection, the expression level of each related gene, including chIFN-α, chIFN-β, chMx, chPKR, chOAS, and chRNaseL, was measured in triplicate by rRT-PCR. The results for the cellular RNA samples were obtained at 0, 6, 12, 24, and 48 hpi. After H1N1 virus infection, the pre-immune state induced by chIFN-γ showed significantly increased expression of chIFN-α, chIFN-β, and chRNaseL mRNA (Fig. 4a, 4b, and 4f). Unlike other genes, chMx peaked early after H1N1 infection at 12 hpi then declined, compared to that in the mock-transfected control (Fig. 4c). However, there was no statistical difference in case of chPKR gene, which showed a similar tendency in both chIFN-γ-transfected CEFs and mock-transfected control cells (Fig. 4d). After H9N2 infection, the pre-immune state induced by chIFN-γ stimulated significantly increased gene expression of chIFN-α, chIFN-β, chMx, and mRNA (Figs. 5a, 4b, and 4c). ChIFN-α and chIFN-β genes exhibit an induction pattern similar to that of H1N1 infection. The only difference was that early expression level (6 and 12 hpi) of chIFN-β was slightly lower than that in H1N1 infection. Compared to the mock-transfected control, chMx was up-regulated with a significant increase, which was maintained until 24 hpi (Fig. 5c). Unlike that observed with H1N1 infection, chIFN-γ did not stimulate higher expression of chOAS and chRNaseL. Likewise, expression of chPKR was not markedly high compared to that observed in the mock-transfected control.

## Discussion

The role of IFN-γ and antiviral proteins against influenza virus is highlighted by the studies; 1) Pretreated human IFN-γ inhibit virus replication in St. Jude porcine lung epithelial cell, 2) Influenza virus encodes a non-structural protein 1(NS1) designed to resist antiviral properties of PKR and OAS/RNase L [22, 23]. In a previous study, we had demonstrated that immunocompromised chickens with decreased chIFN-γ mRNA expression in blood show relatively higher mortality than normal chickens after H9N2 infection [13]. Taken together, we supposed that upregulation of chIFN-γ could be one of the key factors that need to be disrupted for successful infection of influenza virus. Therefore, we hypothesized that the pre-immune state induced by IFNs would decrease influenza virus infection, which could prevent the host from failure to induce a cell-mediated antiviral response. Although few studies tried to understand the pre-treatment effects of IFNs, none of them focused on chIFN-γ-induced antiviral response against AIV infection in CEFs.

CEFs are a useful tool for studying the interactions between avian viruses and immune-related genes. Because viral infection of cell culture systems can be controlled, specific molecular and cellular changes that occur in the host cells early during the course of the infection can be analyzed. Therefore, the response of cell culture systems to influenza has been characterized in many ways [24–26]. Compared to other expression systems, the chIFN-γ produced by CEFs showed a different molecular size varying from 13 to 24 kDa [27, 28]. Two major bands were observed, one of which corresponds to the theoretically calculated size of chIFN-γ, while the other one was higher than the expected size. C-terminus truncation might have occurred, as observed when chIFN-γ was expressed in *Escherichia coli* [29]. The bands located above the major bands could be produced by glycosylation, three possible sites for which are present on chIFN-γ. When we observe the immunofluorescence imaging, the overall transfection efficiency of the chIFN-γ was about 25 %. Because cell growth inhibition and apoptosis are one of the effects of IFN-γ, we controlled the transfection efficiency under the 30 %, which did not affect the cell health [30].

Immediate early induction of some ISGs in the CEFs after H1N1 and H9N2 virus infection is well characterized by Sutejo et al. [24]. To the best of our knowledge, however, no previous studies have studied how the chIFN-γ-induced pre-immune state affects antiviral gene expression after influenza infection in CEFs. In our study, pretreatment of chIFN-γ lead to elevation of chIFN-α and chIFN-β mRNA levels. This is consistent with previous studies [30], indicating that the induction of IFN-γ leads to cross-talk between the IFN-α and IFN-β pathways. However, the approach used here differs in terms of the chIFN-γ expression system of CEFs, not in the addition of recombinant chIFN-γ to the cell culture medium. In addition, we observed the regulation of ISGs, which is related to the antiviral effect against influenza infection. We also found that because fibroblasts are a major cellular source of IFN-β, the fold increase in chIFN-β was higher than that in chIFN-α in both H1N1 and H9N2. [31]

ChMx expression markedly surged early after the infection, supporting previous findings [32]. However, it was previously reported that transfected cells expressing chMx do not lead to enhanced resistance to influenza virus [33]. An additional study was performed to understand the polymorphisms of the chMx protein that lead to alterations in antiviral activity [34]. Notably, the elevation of chMx mRNA was faster than that of other ISGs. These data suggest that the pre-immune state induced by chIFN-γ in CEFs led to rapid induction of chMx. This may play a key role in the observation that H1N1 and H9N2 virus infected into chIFN-γ-transfected CEFs had an approximately 17 and 2.2-fold reduced HA gene replication rate compared to the virus infected into the mock-transfected control cells. The finding that the peak time of expression was shorter in H1N1 infection than in H9N2 infection was unexpected. Unfortunately, we cannot directly compare our findings with those of previous studies because we used different viral strains and cells. However, there is evidence that suggests that infection with H9N2 of avian origin induces more Mx gene than does H1N1 of human origin [24]. It should be further confirmed that this observation reflects the specific property of each virus, to further understand the sequence of events during the early phase of infection.

As reported previously [35], the IFN-induced antiviral proteins chOAS/RNaseL and chPKR are up-regulated as part of the host protective response in chickens infected with the AIV subtype H9N2. In this study, although the expression of chPKR was not significantly up-regulated in the CEFs transfected with pcDNA-chIFN-γ, the increase in the levels of chOAS and chRNaseL suggests that the antiviral status was induced by the pre-immune state after H1N1 infection, but not after H9N2 infection. Usually, PKR and OAS are activated upon detection of double-stranded vRNA, leading to arrest of translation and inhibition of viral protein synthesis [36]. The pre-immune state stimulated by chIFN-γ did not induce increased expression of chPKR and chOAS, compared to the mock-transfected control cells. Although the increase of chOAS was higher in H1N1 infection than in H9N2 infection, because it was observed at 48 hpi, it cannot support the marked increase of chRNaseL after H1N1 infection. Although the viral dsRNA involved in activating chOAS acted similarly in H1N1 and H9N2 virus infections, the H9N2 virus derived from avian species could be more efficient in sequestering viral dsRNA from chOAS by NS protein in the CEFs [37].

To investigate the effects of chIFN-γ-induced pre-immune state, the released H1N1 and H9N2 viral particles were measured in the CEF culture medium with and without chIFN-γ activity. Although the decrease in virus replication was minor, the chIFN-γ-producing CEFs can reduce virus replication in H1N1 and H9N2 infection. Our backward approach involved the thought that the suppression of chIFN-γ with siRNA increases virus gene replication in H1N1 and H9N2 infection in the CEFs. This reduction in chIFN-γ levels was accompanied by an increase in the expression of viral HA and PB2 genes in chIFN-γ-siRNA 277 bp-transfected CEFs.

## Conclusions

In this study, an expression system was used to stimulate the intrinsic cellular defense mechanism in CEFs by using cloned chIFN-γ. Overall, our data showed that the pre-immune state induced by chIFN-γ in CEFs successfully develops intrinsic immunity by enhancing the expression of ISGs such as chMx, chOAS, and chRNaseL. Importantly, these ISGs have been reported to exert antiviral activities against influenza infection. These results suggest that stimulating innate immunity before the infection prevents virus replication and inhibits the release of viral progeny. We believe that our results can contribute to improved understanding of the mechanism underlying the preventive effect exerted by chIFN-γ against influenza.

## Ethics approval and consent to participate

All animal procedures performed in this study were reviewed, approved, and supervised by the Institutional Animal Care and Use Committee (IACUC) of Konkuk University (no. KU13178).

## Availability of data and materials

The datasets supporting the conclusions of this article are included within the article and its additional file.

## Additional files

Additional file 1: Table S1. Genes, GenBank Accession numbers, and Primers used in Real-time RT-PCR. (DOCX 14 kb) Additional file 2: CEFs were transfected with 50nM and 10nM concentration of siRNA targeting chIFN-γ. The cells were harvested at 12 and 24 h post transfection and analyzed by rRT-PCR. Relative expression levels of chIFN-γ mRNA was normalized and calculated using the comparative 2^-2∆∆*Ct*^ method. Error bars are standard deviation of the average. Asterisk represent significance when compared to control siRNA transfection (*P* < 0.001). (DOCX 51 kb)

## Abbreviations

AIV: Avian influenza virus
CEFs: Chicken embryo fibroblasts
chIFN-α: Chicken interferon alpha
chIFN-β: Chicken interferon beta
chIFN-γ: Chicken interferon gamma
chMx: Chicken myxovirus-resistance protein GTPase
chOAS: Chicken oligo-adenylate synthetase
chPKR: Chicken RNA-dependent protein kinase
chRNaseL: Chicken latent ribonuclease
CPE: Cytopathic effect
FBS: Fetal bovine serum
HA: Haemagglutinin
IFN: Interferon
IFN-γ: Interferon gamma
ISGs: Interferon-stimulated genes
MDCK: Madin-Darby canine kidney
MEM: Minimum essential medium
mRNA: messenger RNA
Mx: Myxovirus-resistance protein GTPase
OAS: Oligo-adenylate synthetase
PB2: Polymerase basic 2
PBMCs: Peripheral blood mononuclear cells
PKR: RNA-dependent protein kinase
RNaseL: Latent ribonuclease
siRNA: Small interfering RNAs
SPF: Specific pathogen free
vRNA: viral RNA
