# Supplementary material for: Pre-immune state induced by chicken interferon gamma inhibits the replication of H1N1 human and H9N2 avian influenza viruses in chicken embryo fibroblasts
Source: Virol J. 2016 Apr 27;13:71. doi: 10.1186/s12985-016-0527-1 (PMC4847267; doi:10.1186/s12985-016-0527-1)
Supplement: Additional file 1: Table S1. — Genes, GenBank Accession numbers, and Primers used in Real-time RT-PCR. (DOCX 14 kb) [file 12985_2016_527_MOESM1_ESM.docx]

Additional file 1: Table S1

Genes, GenBank Accession numbers, and Primers used in Real-time RT-PCR

| Gene (GenBank Acc. No.) | Sense primer(5’→3’) | Antisense primer(5’→3’) |
| --- | --- | --- |
| chIFN-α(X92476) | TCCAAGACAACGATTACAGCGCCT | TGTTGCCTGTGAGGTTGTGGATGT |
| chIFN-β(NM_001024836 ) | ACCTTCTCCTGCAACCATCTTCGT | ATGGCTGCTTGCTTCTTGTCCTTG |
| chIFN-γ(AY501004.1) | AACTGAAGAACTGGACAGAGAG | GTGTTTGATGTGCGGCTTTG |
| chMx(AY695797 ) | ACTGACTGACAGAAAGCCTGAGCA | CAATTGCAGGCAACATCAGGTCGT |
| chRNaseL(NM_001031267 ) | GCACTGTGTTTATTGAGGCAGCCA | TCCCATACCAAGCAGCTTCCATGA |
| chOAS(AB037592) | CACGGCCTCTTCTACGACA | TGGGCCATACGGTGTAGACT |
| chPKR(AB125660) | TGGTGTGAAGTATGGTACAGGCGT | TGCTTTGGCTCAATCATGTCCCAC |
| chGAPDH(NM_204305) | CCCAGCAACATCAAATGGGCAGAT | TGATAACACGCTTAGCACCACCCT |
| H9N2 PB2(AY862718) | TTTGACTCAAGGAACCTGCTGGGA | ACCATGACACATCTCCAAGAGCGA |
| H9N2 HA (AY862606) | ACCAAATACAGGACATCTGGGCGT | TGAACCCAATGCTCTCTTCACCTT |
| H1N1 PB2 (CY009611) | AGAGACGAACAGTCGATTGCCGAA | ATCGCTGATTCGCCCTATTGACGA |
| H1N1 HA (CY009604) | ACTCACTGCTTCCAGCGAGATCAT | TTGTGGTTGGGCCATGAACTTTCC |
