# Supplementary material for: Pre-immune state induced by chicken interferon gamma inhibits the replication of H1N1 human and H9N2 avian influenza viruses in chicken embryo fibroblasts
Source: Virol J. 2016 Apr 27;13:71. doi: 10.1186/s12985-016-0527-1 (PMC4847267; doi:10.1186/s12985-016-0527-1)
Supplement: Additional file 2: — CEFs were transfected with 50nM and 10nM concentration of siRNA targeting chIFN-γ. The cells were harvested at 12 and 24 h post transfection and analyzed by rRT-PCR. Relative expression levels of chIFN-γ mRNA was normalized and calculated using the comparative 2-2∆∆Ct method. Error bars are standard deviation of the average. Asterisk represent significance when compared to control siRNA transfection (P < 0.001). (DOCX 51 kb) [file 12985_2016_527_MOESM2_ESM.docx]

Additional file 2. CEFs were transfected with 50nM and 10nM concentration of siRNA targeting chIFN-$\gamma$. The cells were harvested at 12 and 24 hour post transfection and analyzed by rRT-PCR. Relative expression levels of chIFN-$\gamma$ mRNA was normalized and calculated using the comparative $2^{-\Delta\Delta Ct}$ method. Error bars are standard deviation of the average. Asterisk represent significance when compared to control siRNA transfection (*P* < 0.001).
